# Supplementary material for: Fast and Fourier features for transfer learning of interatomic potentials
Source: NPJ Comput Mater. 2025 Sep 25;11(1):293. doi: 10.1038/s41524-025-01779-z (PMC12463661; doi:10.1038/s41524-025-01779-z)
Supplement: Supplementary file 1 — Supplementary information [file 41524_2025_1779_MOESM1_ESM.pdf]

Fast and Fourier Features for Transfer Learning of Interatomic Potentials

Supplementary Material

Pietro Novelli, Giacomo Meanti, Pedro J. Buigues,Lorenzo Rosasco,
Michele Parrinello, Massimiliano Pontil, and Luigi Bonati

TM23

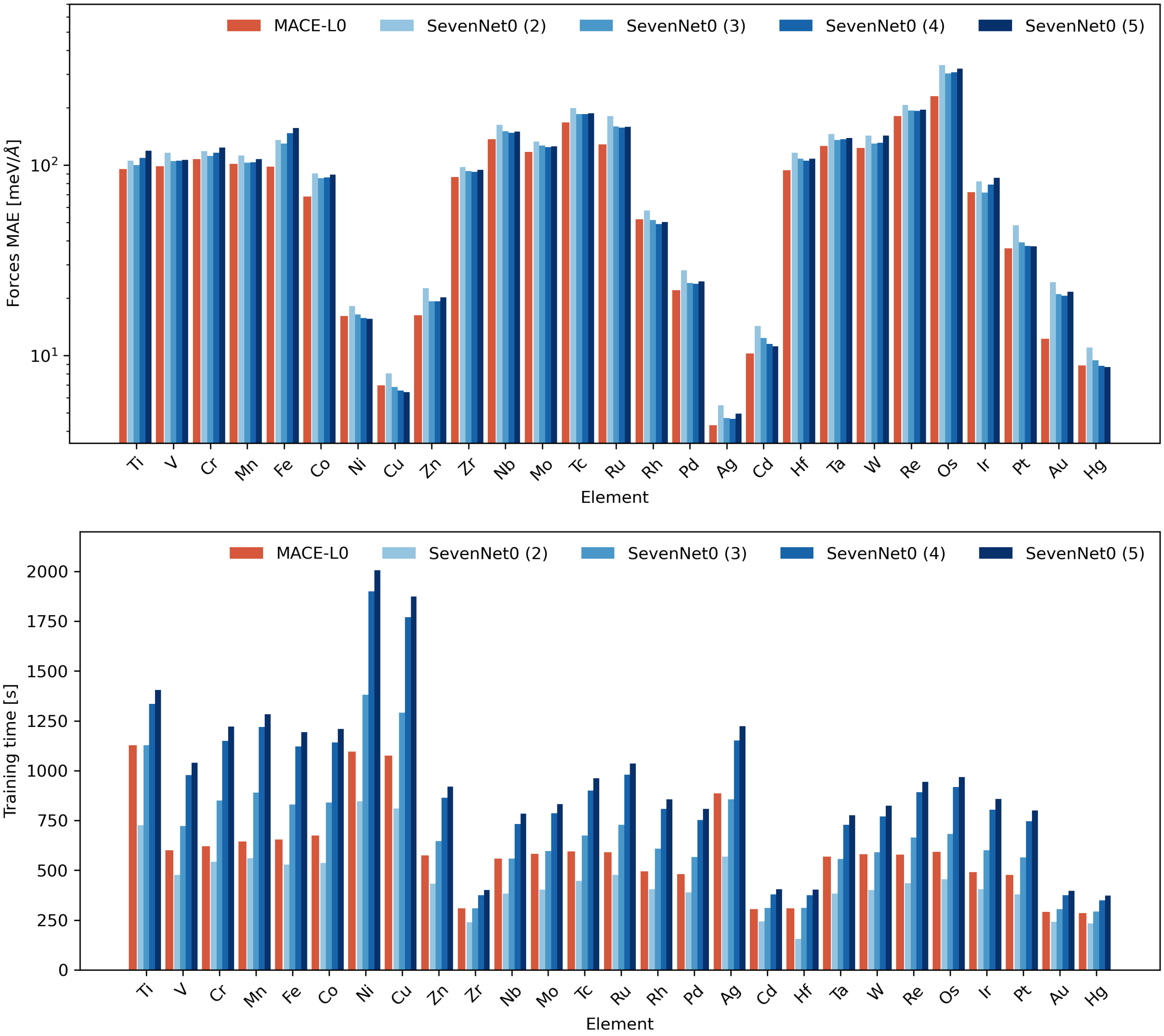

Figure S1. **Forces accuracy and timings for the SevenNet0 backbone**. (top) Forces MAE for each element of the TM23 dataset. (bottom) Training times for the SevenNet0 models. In both panels, each bar represent `franken`model trained using representations extracted at different interactions layer, from 2 to 5. As a baseline, the MACE-MP0 used in the manuscript is shown in red. Note that for the MACE architecture, we do analyze the dependence on the interaction layer since it only has 2 of them. The behavior along the periodic table between the backbones is very similar, although the SevenNet0-based models are slightly less accurate but also faster if a representation from the first layers is used.

Bulk water

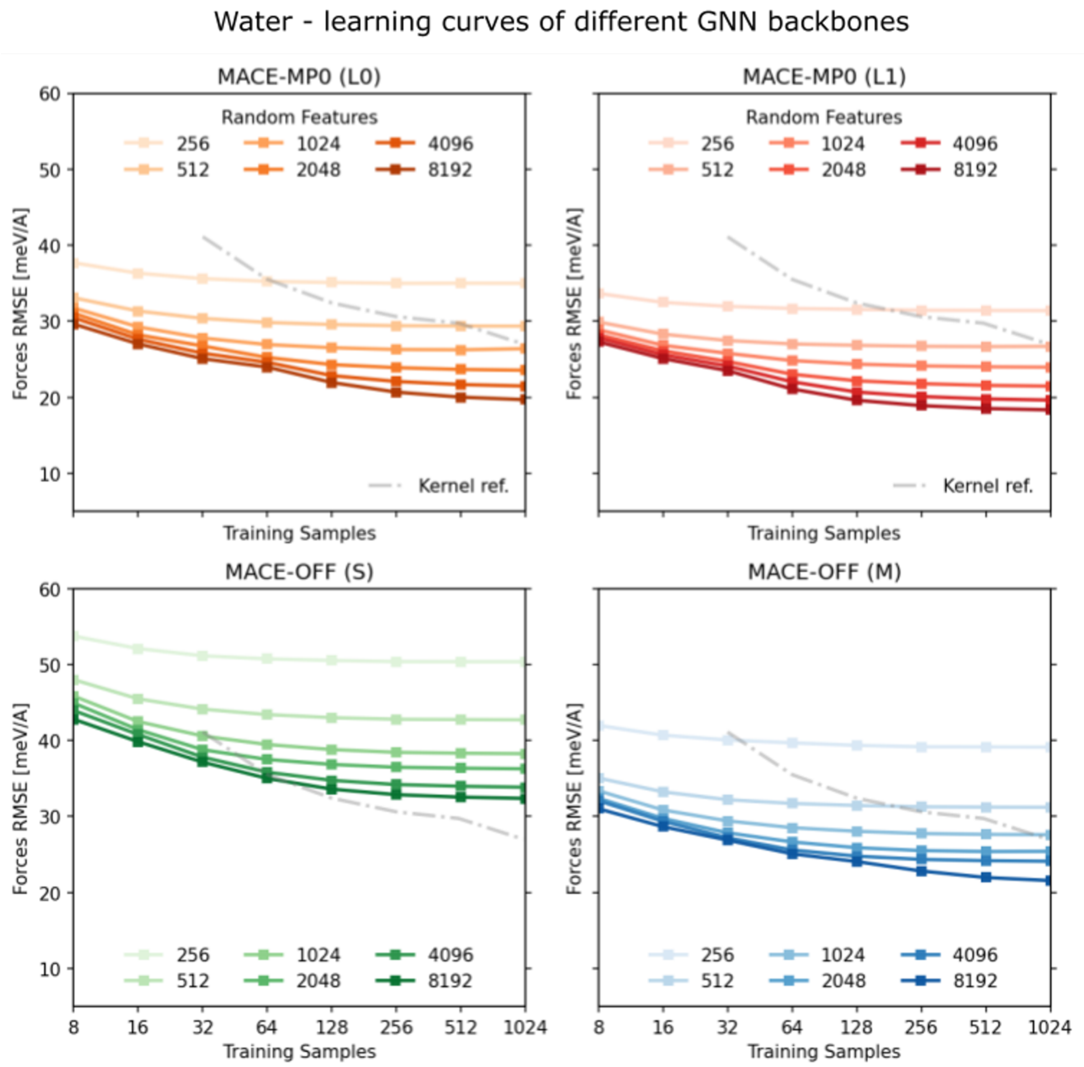

Figure S2. **Sample complexity studies vs different MACE backbones and number of RFs**. Each panel contains the learning curves in terms of the number of samples for four MACE backbones (MACE-MP0-L0 and L1, MACE-OFF small and medium). Each line correspond to a different number of random features, to show the monotonic increase upon model complexity.

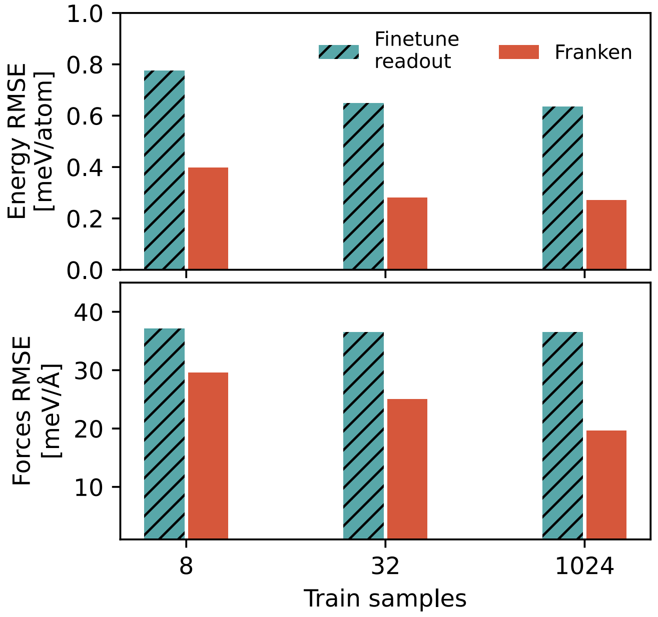

Figure S3. **Comparison with readout finetuning**. The RMSE for energy (top panel) and forces (bottom panel) is shown as a function of the number of training samples (8, 32, 1024) for `franken` and a baseline where the GNN representations are kept frozen and only the original readout layers are optimized. For the fine-tuning baseline, a learning rate of 10^{-3} is used with a batch size of 8 and 2048 gradient update steps.

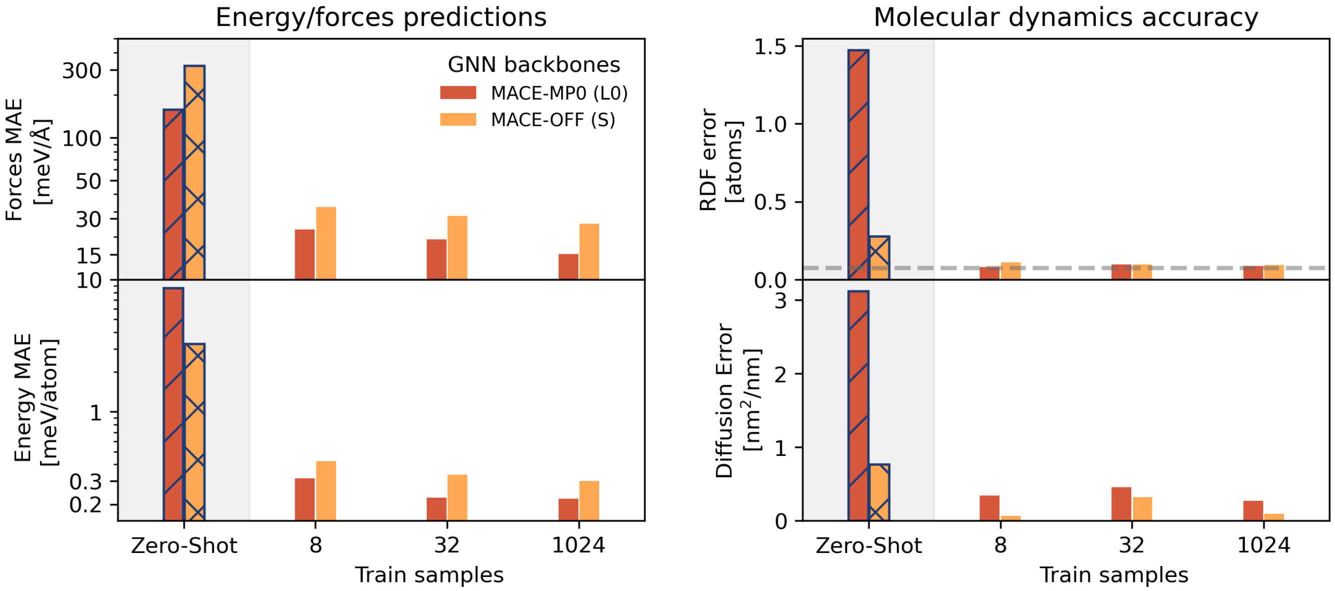

Figure S4. **Sample efficiency on the water dataset** for force and energy predictions (left) and MD properties (right). Same as Fig. 4 but for two different backbones: MACE-MP0 L0, red and MACE-OFF-small in orange. The first columns are related to the zero-shot performances.

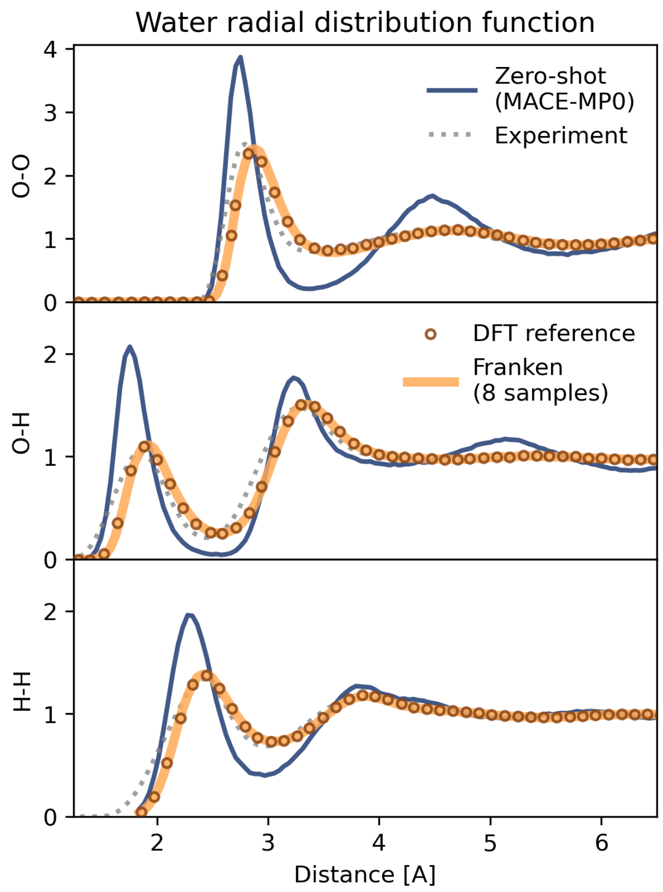

Figure S5. **Partial radial distribution functions for water** for oxygen-oxygen (top), oxygen-hydrogen (center), and hydrogen-hydrogen (bottom). The solid blue line denotes the zero-shot result obtained with MACE-MP0 $L = 0$, while the orange thick line denotes `franken`'s results using the same backbone, 4096 RFs, and trained on 8 configurations. The empty circles represent the DFT reference, and in a dotted grey line, the experimental curve, both taken from Ref. [70].

Table S1. **Inference timings for models trained on different MACE backbones**. Comparative inference time benchmark of the different MACE-MP0 (L0 and L1) and MACE-OFF (small and medium) backbones used in this work for zero-shot and `franken`. Timings were obtained using ASE-based[65] calculators such as the `MACECalculator` as implemented in the MACE[27] Python package for MACE-based models and our custom `FrankenCalculator` implementation for `franken` models predicting energy and forces for a box of water molecules.

		Atoms/ms		
Model		Zero-shot	`franken`	Speedup
MACE-MP0	L0	8.9	11.8	1.32x
	L1	5.4	6.4	1.18x
MACE-OFF	small	16.2	21.5	1.32x
	medium	7.9	9.4	1.2x

Pt(111)/water interface

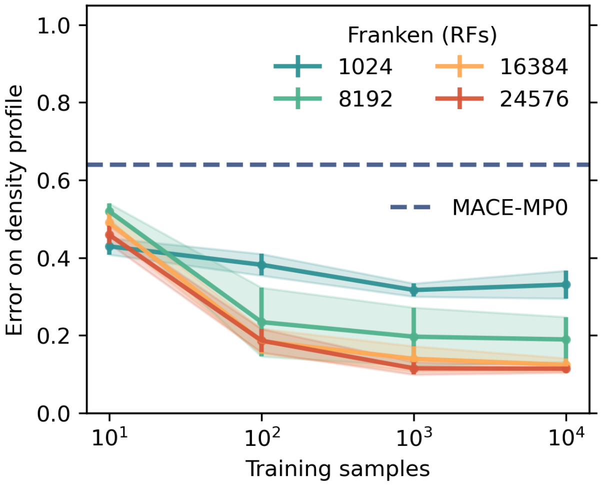

Figure S6. **Data-efficiency of MD observables**. Integrated error on the density profile with respect to the independent reference in Fig. 6 for the zero-shot (MACE-MP0) simulation and the ones obtained with `franken` using different numbers of training samples. Shaded areas indicate standard deviations obtained from 5 independent replicas.
